# Response of spontaneously hypertensive rats to inhalation of fine and ultrafine particles from traffic: experimental controlled study

**DOI:** 10.1186/1743-8977-3-7

**Published:** 2006-05-15

**Authors:** Ingeborg M Kooter, A John F Boere, Paul HB Fokkens, Daan LAC Leseman, Jan AMA Dormans, Flemming R Cassee

**Affiliations:** 1 Centre for Environmental Health Research, National Institute for Public Health and the Environment, Bilthoven, The Netherlands; 2 Laboratory of Toxicology, Pathology and Genetics, National Institute for Public Health and the Environment, Bilthoven, The Netherlands

## Abstract

**Background:**

Many epidemiological studies have shown that mass concentrations of ambient particulate matter (PM) are associated with adverse health effects in the human population. Since PM is still a very crude measure, this experimental study has explored the role of two distinct size fractions: ultrafine (<0.15 μm) and fine (0.15- 2.5 μm) PM. In a series of 2-day inhalation studies, spontaneously hypersensitive (SH) rats were exposed to fine, concentrated, ambient PM (fCAP) at a city background location or a combination of ultrafine and fine (u+fCAP) PM at a location dominated by traffic. We examined the effect on inflammation and both pathological and haematological indicators as markers of pulmonary and cardiovascular injury. Exposure concentrations ranged from 399 μg/m^3 ^to 3613 μg/m^3 ^for fCAP and from 269μg/m^3 ^to 556 μg/m^3 ^for u+fCAP.

**Results:**

Ammonium, nitrate, and sulphate ions accounted for 56 ± 16% of the total fCAP mass concentrations, but only 17 ± 6% of the u+fCAP mass concentrations. Unambiguous particle uptake in alveolar macrophages was only seen after u+fCAP exposures. Neither fCAP nor u+fCAP induced significant changes of cytotoxicity or inflammation in the lung. However, markers of oxidative stress (heme oxygenase-1 and malondialdehyde) were affected by both fCAP and u+fCAP exposure, although not always significantly. Additional analysis revealed heme oxygenase-1 (HO-1) levels that followed a nonmonotonic function with an optimum at around 600 μg/m^3 ^for fCAP. As a systemic response, exposure to u+fCAP and fCAP resulted in significant decreases of the white blood cell concentrations.

**Conclusion:**

Minor pulmonary and systemic effects are observed after both fine and ultrafine + fine PM exposure. These effects do not linearly correlate with the CAP mass. A greater component of traffic CAP and/or a larger proportion ultrafine PM does not strengthen the absolute effects.

## Background

Epidemiological studies have shown that exposure to ambient particulate air pollution (particulate matter or PM) is associated with many health effects [1-3], which include premature death, increased hospitalization for cardiopulmonary diseases, airway complaints, and reduced lung function. Although estimates of relative risks are small, there is a public-health concern because many people are exposed and there are high-risk groups, such as the elderly, very young children, and people with cardiopulmonary diseases. Although the PM-associated adverse health effects have been found all over the world, a more closer look reveals that there seem to be heterogeneous across locations [4-8], which might be due to differences of anthropogenic sources such as traffic [9]. Particulate matter consists of many chemicals, but it is not very likely that some of them (sea salt, sulphate, and nitrate [10] in ambient air affect health adversely. Aerosolized combustion products from traffic, shipping, industry, and domestic heating are believed to be far more relevant. The risk can be effectively reduced by reducing the PM fraction that is most likely to cause adverse health effects.

Due to its the complexity, the best way to study PM is by studying the effects of inhaling it. Systems designed to deliver controlled amounts of concentrated ambient particles now exist and allow a mechanistic approach to determining the effect of inhaled PM in different size ranges [11-13]. Recently published studies have shown that exposing rodents [14-20], dogs [21] and human volunteers [22-26] to concentrated ambient particles (CAP) indicate that PM has the potential to cause adverse effects. Biological responses to high concentrations of PM (which were often well above ambient PM concentrations) were observed. The sensitivity of these toxicological studies is low because of the small number of observations, as well as the fact that exposure levels and PM composition vary from day to day. However, data from homogeneous populations, as well as the use of specific disease models that mimic human risk groups, should increase study sensitivity to the effects of CAP exposures. The results of a series of 1-day (6 h/day) inhalation exposures of compromised rats to fCAP [27] revealed that CAP can increase inflammation [polymorphonuclear leukocytes (PMNs)] and toxicity [protein and albumin in bronchoalveolar lavage fluid (BALF)], and it can also increase the risk of thrombotic vascular disorders (fibrinogen). Nonetheless, we were unable to prove consistent relationships between PM mass and biological effects. While alterations of biological endpoints were occasionally statistically significant and potentially biologically relevant, we found no convincing proof that ambient PM exposures (up to 3500 μg/m^3^) can modify homeostasis. In another study in which rats pre-treated with ozone or with induced pulmonary hypertension were exposed for 6 h to concentrated freshly generated diesel exhaust particles up to 9000 μg/m^3^, no noteworthy pulmonary toxicity was observed, though increased glutathione levels (in the case of ozone-treated rats) and increased blood fibrinogen levels in rats with existing pulmonary hypertension were observed [20]. Although these studies and those of others [14,15] have shown that CAP exposure has adverse effects, no studies have yet been published that convincingly prove relationships between mass concentration and these biologically relevant outcomes [18,27]. Bearing the available evidence of studies exposing animal or human subjects to CAP, PM mass concentrations do not seem to be the optimal metric to be associated with the adverse health effects. In that respect it is noteworthy that monotonic functions are usually sufficient to describe the relationship between air pollution and health effects in the epidemiological studies. However, Seagreave and colleagues [28] have recently shown that at least some parameters may respond in a way that achieves an optimum concentration beyond which the effects are reduced again.

Most toxicological studies that have used CAP exposures have focused on the accumulation mode (0.15 μm – 2.5 μm) [15,17,19] or coarse mode (2.5 μm – 10 μm) [17] of ambient PM. In recent years, more and more information has shown that the ultrafine fraction within PM might be more toxic than the fine mode. It has been suggested that the large surface area or particle number, or perhaps just the particle size, may play an important role in such differential responses [29,30]. Other studies have shown that the ultrafine fraction of urban ambient aerosols is not necessarily more potent than the fine or coarse fractions in inducing inflammatory and toxic effects in lung cells [31].

In order to compare the potential of PM fractions to induce adverse biological effects, we performed a series of studies of spontaneously hypertensive (SH) rats in which we used both concentrated fine PM (fCAP) and ultrafine + fine PM (u+fCAP). We compared the effects of PM that included ultrafines at a site with traffic as the major source with toxicity of accumulation mode (fine PM) at an urban background site with no dominant source of PM emission. A strain of SH rats was selected as a strain that would be more sensitive to PM [19] and to allow interstudy comparison [27,15].

## Results

### Exposure characteristics

Integrated and continuous exposure characterization techniques were applied to determine the concentration and the composition of the test atmospheres at the different exposures (Table 1). Temperature and relative humidity were 22 ± 2°C and 40 ± 10% for the fCAP exposure and both control exposures; they were 22 ± 2°C and 70 ± 10% for the u+fCAP exposure. The mean overall levels of some gaseous pollutants before passing the air through the concentrator were: 12 μg/m^3 ^for ozone, 39 μg/m^3 ^for carbon monoxide, 29 μg/m^3 ^for sulphur dioxide, 19 μg/m^3 ^for nitrogen monoxide, 13 μg/m^3 ^for nitrogen dioxide and 28 μg/m^3 ^for total nitrogen oxides (NOx). Ambient ozone is known to be efficiently removed in the concentrator due to the large metal surface, whereas all other gaseous components will remain at the same concentration or will show a little decrease. Table 1 shows that the sum of ammonium, nitrate, and sulphate ions accounts for 56 ± 16% of the total fCAP mass concentrations for the Bilthoven site (I), whereas it only accounts for 17 ± 6% of the u+fCAP mass concentrations at the HIA site (II). Most of the remaining mass is very likely associated with carbonaceous material, and the difference between site I and site II reflects the enrichment in organic and elemental carbon, as expected, given its proximity to mobile sources.

**Table 1:** Exposure characteristics for studies in spontaneously hypertensive rats exposed to concentrated ambient particulate matter

Exposure		fCAP (Site I = Bilthoven)					u+fCAP (Site II = HIA)							
Date		6-1-03	13-1-03	20-1-03	27-1-03	11-2-03	8-7-03	29-9-03	6-10-03	13-10-03	4-10-04	6-10-04	11-10-04	13-10-04
Mass^1)^	μg/m^3^	1067.5	456.5	399.0	609.5	3613.0	269.0	409.0	366.5	379.0	501.2	448.2	555.8	534.7
Diameter^2)^	μm	0.65	0.74	0.71	0.75	0.71	0.68	0.72	0.75	0.67	1.09	1.41	0.58	1.08
gsd^2)^		1.14	1.34	1.35	1.30	1.17	1.32	1.33	1.56	1.35	0.21	0.28	0.25	0.22
Number counts^2)^							320,000	470,000	66,000	45,000	998,750	1,310,320	1,624,000	1,282,000
*Compounds*^3)^														
SO_4_^2-^	μg/m^3^	240	86	63	98	908	19	27	25	17	14	28	26	41
NO_3_^-^	μg/m^3^	349	86	66	112	860	16	37	16	37	33	50	33	53
NH_4_^+^	μg/m^3^	194	53	40	68	684	8	18	10	22	9	17	11	25
Cl^-^	μg/m^3^	22	51	42	96	65	38	33	45	35	1	1	1	1
*Gases*^4)^														
O_3_	μg/m^3^	22	16	22	22	10	nm	nm	nm	nm	10	0	0	0
CO	μg/m^3^	32	42	32	31	48	nm	nm	nm	nm	39	40	41	37
SO_2_	μg/m^3^	40	42	36	26	12	nm	nm	nm	nm	6	14	14	13
NO	μg/m^3^	6	5	5	5	6	nm	nm	nm	nm	28	32	50	27
NO_2_	μg/m^3^	9	11	9	9	21	nm	nm	nm	nm	17	19	15	17
NO_x_	μg/m^3^	10	12	9	9	13	nm	nm	nm	nm	41	45	55	41

Experiments A-E concentrated PM in size range of 0.15–2.5 μm, F-N in size range <2.5μm including PM<0.15 μm. fCAP, fine concentrated ambient particulate matter; u+fCAP, ultrafine plus fine concentrated ambient particulate matter

1) Time-integrated 2-day mean mass concentrations at the outlet of the particle concentrator

2) Diameter, gsd (geometric standard deviation) and number are measured by an aerodynamic particle sizer which measures only particles larger than 0.5 μm. Results are based on numbers

3) Chemical analyses (ion chromatography) of filter used for mass concentration

4) nm, not measured

### Bronchoalveolar lavage

Table 2 presents the overall results of the BALF biochemical analyses. The contribution to the day-to-day variation was calculated in a two-way ANOVA for the biological indices of both the filtered air and the CAP- exposed animals. Some of the parameters showed substantial day-to-day differences that might be attributed to the assays and need to be taken into account as a confounding factor in the overall statistical analysis. The CAP exposures, with either the combined fCAP exposures (at site I Bilthoven) or the combined u+fCAP exposures (at site II HIA) did not result in statistically significant changes of most biochemical analyses measured in BALF compared to their filter-air controls. No signs of cytotoxicity as indicated by unchanged LDH and ALP levels were observed. No significant changes were found either in the number of cells or cell differentiation in the BALF. However, clear particle uptake was observed in macrophages lavaged from the rat lungs after exposure to fCAP or u+fCAP. MDA, a parameter for lipid peroxidation, is significantly lowered by u+fCAP exposures (Table 2).

**Table 2:** Health effect parameters measured in lung lavage fluid of spontaneously hypertensive rats 8 h afterexposure to concentrated ambient particulate matter or clean air as a control.

		**fCAP (Site I)**					**u+fCAP (Site II)**				
		Control (*n *= 40)		CAPs (*n *= 40)			Control (*n *= 64)		CAPs (*n *= 64)		

**Parameter**	Units	**Mean**	95% CI	**Mean**	95% CI	Sign.	**Mean**	95% CI	**Mean**	95% CI	Sign.
**ALP**	U/L	**57.0**	52.0–62.1	**51.6**	44.9–58.3		**26.7**	23.4–30.0	**26.1**	22.9–29.2	
**LDH**	U/L	**66.3**	62.2–70.4	**67.6**	63.3–72.0		**55.7**	49.5–61.9	**52.5**	50.3–54.8	
**Protein**	mg/L	**314**	277.9–350.4	**349**	295.5–402.2		**274.5**	242–308	**282.0**	256–288	
**NAG**	U/L	**1.31**	1.20–1.43	**1.43**	1.32–1.54		**0.79**	0.70–0.87	**0.81**	0.74–0.88	
**UA-B**	umol/L	**0.29**	0.22–0.35	**0.45**	0.26–0.63		**0.46**	0.36–0.56	**0.42**	0.34–0.51	
**Total Glut.**	umol/L	**0.92**	0.789–1.058	**1.00**	0.836–1.165		**1.34**	1.02–1.65	**1.22**	1.12–1.31	
**GSH**	umol/L	**0.155**	0.099–0.211	**0.244**	0.158–0.329		**0.39**	0.21–0.57	**0.31**	0.22–0.40	
**GSSG:GSH**	ratio	**2.61**		**1.70**			**4.8**		**2.7**		
**TNF-α **	ng/ml	**46.4**	42.5–50.2	**47.7**	43.2–52.1		**45.9**	37.6–54.2	**46.4**	37.9–54.9	
**MIP-2**	ng/ml	**333**	321–344	**311**	297–325		**192.4**	183–202	**192.2**	184–200	^a^
**IL-1b**	ng/ml	**268**	237–299	**248**	210–286		**n.m.**		**n.m.**		
**CC16**	ng/ml	**1.78**	1.41–2.14	**1.98**	1.56–2.40		**3.31**		**3.31**		
**MDA**	μmol/l	**0.268**	0.200–0.335	**0.237**	0.201–0.273		**0.407**	0.341–0.473	**0.341**	0.281–0.401	*
**HO-1**	ng/ml	**0.393**	0.296–0.491	**0.521**	0.347–0.696		**0.517**	0.418–0.615	**0.664**	0.547–0.781	**
**Macrophages**	%	**90.5**	89.2–91.8	**88.7**	87.2–90.1		**94.4**	93.1–95.6	**94.2**	93.0–95.3	
**Neutrophils**	%	**4.95**	3.70–6.20	**4.76**	3.54–5.99		**1.95**	1.26–2.64	**1.95**	1.37–2.54	
**Eosinophils**	%	**0.60**	0.44–0.76	**0.44**	0.28–0.61		**0.42**	0.23–0.61	**0.34**	0.21–0.46	
**Lymfocytes**	%	**1.05**	0.83–1.27	**1.43**	1.08–1.77		**2.60**	1.93–3.28	**2.99**	2.11–3.88	
**Cell count**	*10e^3^	**541**	481–600	**528**	473–583		**469**	381–556	**417**	349–485	
**Macrophages**	*10e^3^	**489**	435–543	**461**	415–507		**445**	358–532	**394**	327–461	
**Neutrophils**	*10e^3^	**25.70**	17.2–34.2	**26.80**	17.8–35.9		**7.88**	4.36–11.41	**7.39**	5.23–9.52	
**Eosinophils**	*10e^3^	**3.04**	2.24–3.84	**2.60**	1.47–3.73		**2.02**	0.73–3.31	**1.46**	0.80–2.11	
**Lymfocytes**	*10e^3^	**5.50**	4.20–6.84	**7.70**	5.60–9.81	P = 0.06	**10.41**	7.43–13.40	**11.87**	8.01–15.73	

**p < 0.01

^a^*n *= 32

ALP, alkaline phosphatase; LDH, lactate dehydrogenase; NAG, N-acetyl glucosaminidase; UA, uric acid, Total Glut., total glutathione; GSH, reduced glutathione;GSSG, oxidized glutathione; TNF-α, tumor necrotic factor; MIP, macrophage inhibiting factor;IL, interleukine, CC16, Clara cell protein; MDA, malondialdehyde; HO-1, heme oxygenase; CI, confidence interval; fCAP, fine concentrated ambient particulate matter; n.m., not measured; u+fCAP, ultrafine plus fine concentrated ambient particulate matter.

Levels of the overall HO-1 values in BALF, as a measure for oxidative stress in the lungs, were increased by u+fCAP and fCAP exposures (after omitting data of the two experiments with the highest exposure concentrations, e.g. January 6^th ^2003 and February 11 2003) (Table 2). For the u+fCAP exposure, there was a significant increase to 0.664 ng/ml compared to the 0.517 ng/ml value of the control animals. However, more detailed data analyses of the fCAP data shows a clear nonmonotonic concentration-effect relationship with the HO-1. Maximum levels of HO-1 were observed at around 600 μg/m^3 ^measured both in BALF and in lung homogenate (Figure 2). There is a correlation of *r*^2^= 0.79 for the HO-1 measured in BALF and in lung homogenate (Figure 3). Data corrected for the amount of protein present were very comparable to Figure 2 (data not shown). In none of the fCAP exposures did the LDH content in BALF change significantly upon exposure.

The only other parameter which showed significance at the individual fCAP exposures was CC16, which was significantly decreased at 457 μg/m^3 ^fCAP and increased at the greatest exposure of 3613 μg/m^3 ^fCAP (Figure 4).

### Blood

After exposure to fCAP and u+fCAP, there was a significant decrease of WBCs (Table 3). Consequently, absolute numbers of neutrophils and lymphocytes are decreased (although the decrease is not statistically significant for the number of neutrophils after u+fCAP exposure). Significant changes in haematological parameters, i.e., the mean platelet volume (MPV) and the mean platelet component (MPC), were found after exposure to u+fCAP, but these effects are not observed for the fCAP-exposed rats (the MPC parameter has not been measured for the fCAP exposures). No changes in concentrations of fibrinogen and vWF were observed in plasma after exposure to u+fCAP or fCAP. However, a significant increase of the greatest concentration of the fCAP exposure was observed for vWF (Figure 5).

**Table 3:** Health effect parameters measured in blood of spontaneously hypertensive rats 8 h after exposure to concentrated ambient particulate matter or clean air as a control.

		**fCAP (Site I)**					**u+fCAP (Site II)**					
		Control (*n *= 40)		CAP (*n *= 40)			Control (*n *= 32)		CAP (*n *= 32)			Variation among

		Control (*n *= 40)		CAP (*n *= 40)								
**Marker**	Units	Mean	95% CI	Mean	95% CI		Statistical significance	95% CI	Mean	95% CI		experiments
**WBC**	^* ^e9/L	**3.20**	3.01–3.38	**2.89**	2.68–3.10	*	**3.82**	3.61–4.02	**3.51**	3.31–3.72	*	***
**RBC**	^* ^e12/L	**8.67**	8.59–8.76	**8.74**	8.66–8.82		**8.88**	8.78–8.97	**8.94**	8.86–9.03		***
**HGB**	mmol/L	**8.69**	8.60–8.77	**8.75**	8.67–8.82		**8.66**	8.56–8.76	**8.69**	8.60–8.78		***
**HCT**	L/L	**0.404**	0.400–0.408	**0.408**	0.404–0.412	*	**0.42**	0.410–0.419	**0.42**	0.411–0.418		***
**MCV**	fL	**46.61**	46.22–46.99	**46.74**	46.35–47.14		**46.72**	46.53–46.91	**46.33**	46.13–46.53	**	
**MCH**	fmol	**1.002**	0.994–1.010	**1.002**	0.995–1.010		**0.98**	0.971–0.980	**0.97**	0.967–0.976		**
**MCHC**	mmol/L	**21.50**	21.42–21.58	**21.42**	21.35–21.50		**20.90**	20.82–20.94	**20.97**	20.90–21.04	*	***
**RDW**	%	**11.83**	11.69–11.98	**11.68**	11.55–11.80		**11.64**	11.53–11.76	**11.67**	11.51–11.82		
**HDW**	mmol/L	**1.44**	1.411–1.4778	**1.44**	1.408–1.474		**1.52**	1.506–1.539	**1.53**	1.515–1.544		*
**PLT**	^* ^e9/L	**942**	913–972	**929**	899–959		**992**	964–1019	**992**	946–1038		***
**MPV**	fL	**7.39**	7.29–7.50	**7.38**	7.31–7.46		**7.10**	7.01–7.19	**7.24**	7.16–7.32	***	***
**MPC**							**23.30**	23.06–23.56	**23.86**	23.61–24.11	^a ^***	***
**Reticulo-**	abs	**75.8**	71.3–80.4	**72.0**	66.6–77.5		**75.8**	71.3–80.4	**72.0**	66.6–77.5		
**cytes**	%	**0.87**	0.77–0.96	**0.71**	0.64–0.78	*	**0.86**	0.815–0.92	**0.81**	0.75–0.87		*
**Neutrophils**	abs	**0.42**	0.39–0.46	**0.33**	0.31–0.36	***	**0.51**	0.47–0.55	**0.47**	0.44–0.52		*
**Lymphocytes**	abs	**2.65**	2.48–2.83	**2.45**	2.26–2.65	*	**3.13**	2.96–3.30	**2.87**	2.69–3.04	**	***
**Monocytes**	abs	**0.075**	0.067–0.083	**0.0687**	0.060–0.077		**0.103**	0.092–0.114	**0.098**	0.0869–0.109		***
**Eosinophils**	abs	**0.0262**	0.023–0.029	**0.0262**	0.022–0.029		**0.013**	0.0073–0.0190	**0.013**	0.0098–0.0165		***
**Basophils**	abs	**0.00498**	0.00347–0.00649	**0.00517**	0.00381–0.00653		**0.060**	0.0496–0.0699	**0.062**	0.0506–0.0736		***
**LUC**	abs	**0.0129**	0.0109–0.0149	**0.0119**	0.0100–0.0137		**0.008**	0.0043–0.0107	**0.009**	0.0054–0.0117		***
**Neutrophils**	%	**13.54**	12.21–14.88	**11.90**	10.82–12.98	*	**13.37**	12.55–14.20	**13.48**	12.44–14.51		
**Lymphocytes**	%	**82.62**	81.22–84.02	**84.23**	83.11–85.36	*	**81.90**	80.98–82.76	**81.40**	80.23–82.58		
**Monocytes**	%	**2.39**	2.14–2.63	**2.33**	2.15–2.52		**2.66**	2.46–2.87	**2.72**	2.51–2.93		***
**Eosinophils**	%	**0.836**	0.754–0.918	**0.935**	0.823–1.048		**0.340**	0.204–0.475	**0.353**	0.276–0.430		***
**Basophils**	%	**0.203**	0.169–0.236	**0.192**	0.161–0.223		**1.550**	1.33–1.79	**1.790**	1.47–2.12		***
**LUC**	%	**0.384**	0.340–0.427	**0.391**	0.339–0.443		**0.218**	0.152–0.285	**0.254**	0.190–0.318		***
**Fibrinogen**	mg/ml	**2.18**	2.13–2.22	**2.16**	2.13–2.19		**2.236**	2.199–2.274	**2.267**	2.232–2.303	^b^	
**vWF**	ratio	**1.49**	1.34–1.65	**1.64**	1.36–1.92		**0.67**	0.65–0.70	**0.65**	0.61–0.69	^a^	

^a^*n *= 32; ^b^*n *= 61; ^c^*n *= 63; *p < 0.05; **p < 0.01; ***p < 0.001. For abbreviations of the markers, see Materials and methods

### Pathology

There were no differences in the lung and body weights of sham and CAP-exposed animals (data not shown). The characteristic hallmarks of the strain of rats used as controls were clearly present in their lungs: small alveolar haemorrhages and extensive bronchus-associated lymphoid tissue (BALT) at many bifurcations of the airways. These signs were not affected by the CAP exposures (Table 4).

**Table 4:** Summary of histological lung changes due to 2-day filtered air control or exposure to concentrated ambient particulate matter

	**uCAP (Site I)**		**u+fCAP (Site II)**	
Parameter	Control (*n *= 40)	CAP (n = 40)	Control (*n *= 64)	CAP (*n *= 64)
Alveolar macrophages				
minimal	29	33	33	41
slight	0	3	2	2
Foci thick septa + macrophages				
minimal	22	23	31	28
slight	2	0	2	5
Perivascular infiltrate	29	22	51	58
Foci interstitial pneumonia with alveolitis (macrophages + lymphocytes)				
minimal	0	0	1	0
Peribronchitis+hypertrophy bronchial epithelium	0	0	0	1
Macrophages loaded with Particulate matter	0	0	0	52
Erythrocytes in alveoli	28	24	41	39
BrdU score number examined	40	39	64	64
Minimal	27	28	39	35
Slight	1	0	5	4

BrdU, bromodeoxyuridine; CAP, concentrated ambient particulate matter;

No noticeable pathological changes could be observed for either the fCAP or the u+fCAP exposure. Deposition of PM was noted in most of the animals exposed to u+fCAP (52 of 64). This was not observed for animals exposed to fCAP only. Groups of alveolar macrophages were present in nearly all CAP-exposed animals, as in controls.

The lymphocytes in the BALT areas and the perivascular infiltrate of all SH rats were fairly strongly labelled as noted by BrdU incorporation in the DNA, and there was a slight labelling in the alveolar area, which reflects the background turnover. There was slightly more BrdU labelling in all components of those areas of the H&E slides where inflammatory foci were present: an increased proliferation rate in the bronchiolar and alveolar epithelium, as well as in alveolar macrophages. No change of cell proliferation was seen from the labelling-frequency data of nuclei in the control and CAP-exposed groups, and there were no differences between u+fCAP and fCAP groups in this respect (Table 4). The number of foci detected by the immunocytochemical BrdU procedure runs nearly parallel to the observed inflammatory foci with thickened septa in the H&E-stained sections.

## Discussion

Although some epidemiological studies suggest that ultrafine particles have serious health effects [32-34], others fail to prove that this PM fraction is more relevant to health than the fine fraction of PM [35,36], or they fail to separate the effects of ultrafine particles from other air pollutants [37]. The present study focuses on the hypothesis that the ultrafine fraction of PM2.5 dominates the biological responses of rats [32,38,39]. Although there are some small differences between repeated studies with fCAP and u+fCAP, these studies do not fully support our hypothesis.

Besides the normal pathological pulmonary characteristics of the SH rat, no differences (including differences in cell proliferation) were observed between the control rats and the fCAP or u+fCAP exposed rats. However, in contrast to the animals exposed to fCAP (in which more than half of the mass consists of sulphate, nitrate, and ammonium), black particles appeared in the alveolar macrophages of the rats exposed to u+fCAP. This indicates that significant amounts of insoluble particles were deposited in the lungs. It is likely that they originated from combustion processes. Since these exposures were carried out next to a busy freeway, a substantial part of the pollution will have been produced by traffic. Most of the biological endpoints were not affected at all, and the biological relevance of markers that were influenced by CAP exposures remains questionable. This is in line with a previous study [27], in which fCAP (1-day exposures) produced little effect in the bronchoalveolar lavage fluid of SH rats, with the exception of an increase of PMNs.

We added two additional parameters for oxidative stress to the present study: MDA as a measure of lipid peroxidation and HO-1 as a measure of antioxidant response. An increase of lipid peroxidation is expected after exposure to air pollution with oxidative capacity, such as pollution by ozone or PM containing polycyclic aromatic hydrocarbon (PAH) and metals. However, exposure to CAP in the present study resulted in either no significant change (fCAP) or a significant decrease (u+fCAP) of MDA levels in bronchiolar lavage fluid. The biological relevance of these observations is questionable, given the smallness of the decline. Inconsistent data regarding both humans and rats have been reported both as increases [40,41] and decreases [42] of lipid peroxidation after exposure to particles. Where the increase is often seen as a direct result of the oxidative stress, the decrease is assigned to the adaptive capacity of the human organism after prolonged exposure [42].

Exposure to fCAP (after omitting the two highest exposure concentrations) or u+fCAP resulted in a significant increase in HO-1. The enzyme HO-1 is regulated by oxidative stress, catalysing heme oxidation into biliverdin, CO, and iron. The increase results in augmented production of the antioxidant biliverdin and CO, which acts as an anti-inflammatory agent [43]. Indeed, the present study finds no sign of developing inflammation. Various agents, such as endotoxins, cytokines, and heavy metals, as well as CO itself, are known to induce HO-1 [44,45]. The use of HO-1 as a biologically relevant indicator of PM-induced stress has been exemplified in *in vitro *studies in which the PAH content derived from airborne PM positively correlates with increased HO-1 expression [46,29]. It also has been proven that the oxidative potential of CAP in *in vitro *studies correlate well with HO-1 induction [47]. *In vitro *studies that use a murine macrophage cell line [47], have shown that ultrafine PM is a more potent inducer of HO-1 and depleter of intracellular glutathione (anti-oxidant) than ultrafine+fine PM. However, the present study gives no clear indications that, per unit mass, u+fCAP has a greater impact on HO-1 or glutathione than fCAP. Although this might occur at higher exposure levels for u+fCAP than were achieved in this study (550 μg/m^3^), the oxidative stress potency as measured by the HO-1 of ultrafine particles deposited in the lungs may not be significantly greater than that of fine particles.

A statistically significant decrease of WBCs after exposure to fCAP and u+fCAP was noted. There was a concomitant decrease of absolute numbers of neutrophils and lymphocytes. It has previously been reported that these systemic responses, such as a decrease in WBC, are observed 24 h after exposure [25], but not immediately after exposure to CAP [25,26]. Ghio states that the extravasion of neutrophils from the blood into the lung would account for the time dependency of the decrement of the WBC count, since an inflammatory influx after PM exposure is not immediate, but certainly becomes evident at 24 h [25]. However, others [48] report that haematological changes (increase in blood neutrophils and a decrease in lymphocytes 3 h after exposure of rats to CAP (3 h for 110–350 μg/m^3^), but these changes were absent at 24 h after exposure. Such an increase in WBC counts purportedly reflects the inflammatory state after inhalation of PM. In a study similar to ours, Kodavanti et al. [15] report that there were no significant changes in haematological parameters 18–20 h after exposure.

In contrast to previous findings [15,27], we do not observe changes in plasma fibrinogen concentrations as a result of exposure to both u+fCAP and fCAP. The fibrinogen concentration, a risk factor for cardiovascular disease, has been shown to increase after PM exposure in rodent studies [49-51] as well as in human studies [25,52], although decreases have also been observed [49]. A strain-dependent effect in the increase of fibrinogen has also been observed: the effects seem more dominant in SH rats than in Wistar-Kyoto (WKY) rats. This supports the hypothesis that humans with cardiovascular diseases may be more susceptible to increased pulmonary and cardiac impairments [51,53]. A significant increase of fibrinogen levels in SH rats (but not WKY rats) exposed to CAPs has been found [15]. Sela et al [54] proved that oxidative stress resulted in increased plasma MDA, fibrinogen, and PMN counts before hypertension developed in the rat. In this respect, the absence of increased serum fibrinogen levels is rather unexpected. Differences in CAP composition might explain these differences.

The significant changes on the haematological indices corpuscular volume (increased MPV and decreased MCV) in combination with unchanged platelet numbers found after exposure to u+fCAP suggests that the ultrafine particles might have effects on the platelet state. These observations are therefore in agreement with the hypothesis that PM can affect haematological indices [55]. It has been observed that a reduction of MPC may be used to detect *in vitro *platelet activation [56]. However, since the observed differences are very small and opposite in sign, it is questionable whether the changes are biologically relevant.

The fact is that u+fCAP contains less of the soluble inorganic aerosols, sulphates, nitrates, and ammonium than fCAP. Indeed, u+fCAP is very likely to be enriched with carbonaceous (combustion derived) PM since these exposures took place right next to a traffic tunnel. However, except for the black particles in the lung tissue and the changed haematological parameters concerning the platelet state, the effects observed for the fCAP and u+fCAP exposures do not differ.

Studies using concentrated PM are confronted with the fact that air pollution is a complex mixture that varies from day to day. As a consequence, duplication of experiments is virtually impossible. Epidemiological studies have consistently demonstrated that health effects can be predicted with monotonic functions of particle mass concentrations. In turn, CAP studies can be used to verify this relationship under more controlled conditions. In other words, this allows for testing the hypothesis that effects on biological systems due to exposure to PM are always linearly related to the mass concentration. However, the results of the present study indicate that, for example, HO-1 levels show a significant nonlinear relationship with particle mass concentrations. Similar response patterns were seen for CC16 in lavage fluid. In both cases, combined analysis of all experiments did not reveal a statistically significant effect due to CAP exposures. Some other studies report that linear regression shows correlations between a biomarker and the exposure concentrations [27,18,28], but the correlations are usually rather poor. Unfortunately, many of the other markers of biological effect in the present study were not affected at all, which precluded a similar analysis. Therefore, analysis of the combined data of multiple CAPs experiments should always consider that nonmonotonic relationships of concentration effects may be better descriptors of physiological processes. The phenomenon of nonmonotonic relationships of concentration effects applies to many biological processes, for example, enzymatic activity as a function of temperature.

The results of the present series of experiments indicate that minor pulmonary and systemic effects are due to exposure to fine and ultrafine+fine particles at concentrations well above ambient levels. No clear CAP mass correlation has been found for these effects based on the assumption that this relation is linear as shown in epidemiological studies. We even provide evidence that effects due to the oxidative potential of PM might be masked at greater than ambient concentrations, so that prudence is called for when sets of exposures of various concentrations are combined in order to increase the group number of observations in a statistical analysis. In addition, this study shows no proof that change of location, resulting in a larger traffic CAP component, results in noteworthy and biological relevant pulmonary or systemic effects.

## Materials and methods

### Animals

Male SH rats, 11–13 weeks old, were purchased from Charles River Laboratories were assigned to 13 studies (Table 1). Immediately after arrival, the animals were weighed, randomized and then allowed to acclimatize for at least 7 days. The animals were housed in macrolon cages (type III) and fed with SSP-TOX pellets of a cereal-based rodent diet (SMR-A; Hope Farms, Woerden, the Netherlands) and tap water via the automatic drinking-water system, both ad libitum during nonexposure periods. The room temperature was maintained at 22 ± 2°C, the relative humidity at 40–70%, and a 12-h light/dark cycle was maintained.

Each study used eight animals for CAP exposure and eight animals for filtered air exposure (control group). Shortly before exposure, the animals were transported to the mobile exposure laboratory equipped with an ambient particle concentrator, where they were housed in macrolon type III cages, equipped with water bottles. The housing facilities were ventilated with HEPA filters and chemically (activated carbon and purafil) filtered air. Directly after the animals were exposed to CAP or clean air (which was done simultaneously), they were returned to the housing facilities. During exposure, the animals were deprived of water and food.

### Study design

**Figure 1:**
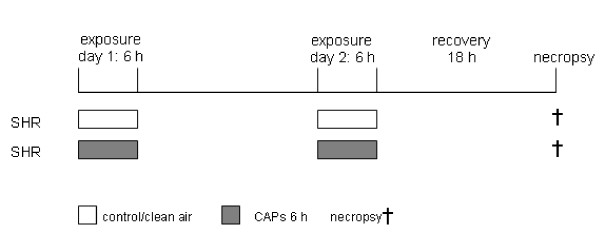
Schematic representation of the study design. Animals were exposed to concentrated ambient particulate matter on day 1 and 2. Necropsy took place 18 h after exposure. SHR, spontaneously hypertensive rat.

A total of 13 studies (which were identical apart from the CAP exposure) were performed at two locations. The SH rats were either exposed to fCAP (five replicate studies located in a city background in Bilthoven = site I) or to u+fCAP (eight replicate studies located in a freeway tunnel near Hendrik Ido Ambacht (HIA; site II)). Each study consisted of a control group of eight animals exposed to HEPA-filtered air, and a group of eight animals exposed to CAP. Exposures lasted 6 h on 2 consecutive days (Figure 1).

### Generation and characterization of the test atmosphere

To obtain fCAP, ambient PM was generated by drawing ambient air through a size selective inlet that removes particles larger than 2.5 μm and subsequently through a four-stage set-up of the ambient fine particle concentrator (AFPC) [11]. The AFPC operates at an air-intake rate of 5000 l/min, and the output flow for PM is 10 l/min. The size distribution of the ambient aerosol after the air passed the PM2.5 size selective inlet was determined during the exposure period with a multi-orifice-impactor (MOI, MSP, Minneapolis, Minn., USA).

The PM mass concentrations were measured continuously at the inlet (ambient) and once an hour (for 5 min) at the outlet of a concentrator during the exposure with a nephelometer (DATARAM, MIE, Billerica, Mass., USA). The time-integrated mass concentrations were also measured at both the inlet and the outlet by means of collection on two 47-mm filters placed in parallel (polytetrafluoroethylene (PTFE) and Quartz), with sampling at a flow rate of 2 l/min. Ozone, carbon monoxide, sulphur dioxide, nitrogen oxides, and the particle number concentrations were recorded every minute in the ambient air behind the PM2.5 impactor. The size distribution of the concentrated aerosol in the range of 0.15 – 2.5 μm was determined once an hour with an aerodynamic particle sizer for particles greater than 0.5 μm (APS, TSI, St. Paul, Minn., USA).

The u+fCAP exposure atmospheres were generated by drawing ambient air through a modified single-stage set-up of a versatile aerosol concentration enrichment system (VACES) [12,13]. The design of the VACES is such that particles greater than 2.5 μm are not concentrated. The VACES operates at an air intake flow rate of 500 l/min, and the output flow for PM is 15 l/min. The temperature of the coolers was kept at a constant -4°C. The temperature of the humidifiers was kept between 25°C and 30°C, depending on the ambient air conditions. The size distribution of the concentrated aerosol in the range of 0.01μm -2.5 μm was determined once an hour with an aerodynamic particle sizer (particles >0.5 μm) (APS, TSI, St. Paul, Minn., USA) for the fCAP experiments.

A condensation particle counter (CPC, TSI, St. Paul, Minn., USA) was used to determine the particle number concentrations after the particles had passed through the concentrator. PM was collected on three 47-mm filters (2 × PTFE and 1 × Quartz) placed in parallel at flow rate of 10 l/min. Similar, PM was collected at a flow rate of 1 l/min after the air passed the concentrator. A carbon sampler tube was placed downstream of one of the PTFE filters at the outlet to collect the VOCs. Ozone, carbon monoxide, sulphur dioxide, nitrogen oxides, and the particle number concentrations were recorded every minute in the ambient air upstream of the humidifier impactor. The size distribution of the concentrated aerosol in the range of 0.02 – 2.5 μm was determined once an hour with an aerodynamic particle sizer (for particles >0.5 μm) (APS, TSI, St. Paul, Minn., USA).

Temperature and relative humidity was recorded once every 5 minutes in the exposure chambers and control exposure chambers, as well as in the ambient air during the exposures. A Sartorius MC-5 microbalance (Sartorius, Goettingen, Germany) was used in controlled relative humidity (40 – 45%) and temperature (22 – 24°C) conditions to do the mass measurements, and the PTFE filters were weighed before and after each field test. Laboratory and field blanks were used for quality assurance. We then analysed the PTFE filters by means of ion chromatography to determine the concentrations of particulate sulphate, nitrate, and ammonium ions. The Quartz filters and activated carbon samplers were stored for future use. At this stage, no efforts have been made for chemical characterization of the PM samples since hardly any effect due to CAP exposure was observed.

### Exposure chamber

Rats were exposed to the test atmosphere in a nose-only exposure chamber placed inside an inhalation unit, which was lighted with tubular fluorescent lamps. The animals were placed in nose-only tubes (Novoplast Tube T, Münster, Muttenz, Switzerland), restrained, and attached to the exposure chamber. The animals could breathe a continuous supply of test atmosphere during exposure (about 8 l/min). Control groups were exposed to air drawn from a concentrator down stream to the size selective inlet, and a HEPA filter filtered the air to provide a particle-free exposure atmosphere. To minimize stress, the animals were allowed to become accustomed to the tubes for 3 days in advance of the exposure, 1 h each day without exposure to the test atmosphere.

### Necropsy

Eighteen hours after exposure, the rats were anaesthetized with Ketamine/Rompun (0.1 ml/100 g body weight of a mix of 0.85 ml 100 mg/ml Ketamine (Aesculaap, Boxtel, The Netherlands) and 0.65 ml of 20 mg/ml Rompun (Bayer, Leverkusen, Germany) and sacrificed by exsanguination via the abdominal aorta. A cannula was inserted in the trachea, and bronchoalveolar lavage fluid (BALF) was taken from the right lung after ligation of the left bronchus. The right lungs were lavaged (three times up and down) with a volume of saline corresponding with 27 ml/kg body weight at 37°C. The fluid recovered from the lavage was placed on ice. The left lung was dissected, weighed, and fixed for 1 h under a constant pressure of 20 cm H_2_O with 10% phosphate-buffered formalin. Five μm paraplast lung sections were stained with haematoxylin and eosin (H&E) and examined under a light microscope.

### Morphometry

To measure cumulative cell proliferation, the animals were injected prior to CAPs exposure and 2 h prior to necropsy with bromodeoxyuridine (BrdU) measured to100 mg/kg body weight (Sigma-Aldrich, Zwijndrecht, The Netherlands). Lung sections from these animals were immunohistochemically stained with anti-BrdU antibody (Boehringer, Mannheim, Germany) and labelled with peroxidase. Since only the inflammatory foci displayed a focally increased proliferation rate, only a semi quantitative labelling score for the size of areas with an increased labelling was assigned. Labelling the frequency in square millimetres of bronchiolar epithelium, for example, makes no sense, as this procedure is fully dependant on how much epithelium is measured both inside and outside an inflammatory focus.

The analySIS soft imaging system (SIS, Münster, Germany) was used to quantify the BrdU-stained cells per millimetre of terminal bronchiolar epithelium. Terminal bronchioles were defined as those bronchioles flowing into alveolar ducts, as well as bronchioles smaller than 250 μm in diameter that are situated in the periphery of the lung and in the close vicinity of a centriacinar area. The total length of examined terminal bronchioles per animal varied between 15 mm and 25 mm.

### Bronchoalveolar lavage analyses

The BALF collected from each animal was centrifuged at 400 *g *and 4°C for 10 min. The cell-free fluid from the lavage was used for biochemical assays. We used a commercial reagent kit (Roche Nederland, Mijdrecht, The Netherlands) to determine the activities of lactate dehydrogenase (LDH), N-acetyl glucosaminidase (NAG), alkaline phosphatase (ALP). The levels of uric acid (UA-B) were determined using a reagent kit obtained from Roche (Almere, the Netherlands) and malondialdehyde (MDA) was determined using a HPLC kit obtained from Chromsystems (Munich, Germany). We determined the total protein levels with a reagent kit obtained from Pierce (Oud-Beijerland, The Netherlands). Methods for the determination of glutathione, both its reduced (GSH) and oxidized (GSSG) forms, and Clara cell secretory protein (CC16) have been described previously [27].

We determined the total cell number by mixing 0.5 ml of the cell suspension with 9.5 ml of Isoton II (Beckman Coulter, Mijdrecht, The Netherlands) and then counting them in a Coulter Counter Z1 and Z2 (Beckman Coulter, Mijdrecht, The Netherlands). For differential cell counts, cytospin preparations were made and stained with the May-Grünwald and Giemsa method. Each cytospin preparation counted 400 cells, and the proportion of each cell type (macrophages, neutrophilic granulocytes, eosinophilic granulocytes, and lymphocytes) was calculated on the basis of total cells per BALF sample.

### Blood analysis

Fibrinogen was determined as a risk factor for thrombotic vascular disorder and von Willibrand factor (vWf) as a marker for early endothelial injury, both as previously described [27].

Cell differentials were determined in ethylenediaminetetraacetic acid (EDTA; Terumo Europe, Leuven, Belgium), and anticoagulated blood was analysed in an H1-E Multi Species Haematology Analyser (Bayer, Mijdrecht, The Netherlands). The following parameters were measured: white blood cell (WBC) and red blood cell (RBC) concentrations, haemoglobin (HGB) and platelet concentrations (PLT), the mean platelet volume (MPV), and the haematocrit value (HCT). The mean corpuscular volume (MCV), mean platelet component (MPC), mean cell haemoglobin (MCH), mean cell haemoglobin concentration (MCHC), red blood cell distribution width (RDW) and haemoglobin distribution width (HDW) are also provided.

### Statistical analysis

All effect parameters were log-transformed before two-way analysis of variance (ANOVA) was performed. Log-transformation is used to account for the increased variation in groups of animals exposed to CAPs versus the animals that were sham exposed. Two-way ANOVA techniques (simple factorial) were used to assess differences due to the factors "CAP exposure", "day-to-day variation" and their interaction while treating "CAP exposure" as a binary term. For those biological parameters that showed a significant effect of "CAP exposure" factor between control animals and CAP-exposed animals, the binary exposure factor was replaced by particle mass CAP as a continuous grouping factor, after which another two-way ANOVA and univariate regression analysis were performed. S-Plus software was used for all statistical analyses. The criterion for significance was set at *p *< 0.05.

## Abbreviations

ANOVA – analysis of variance; ALP – alkaline phosphatase; BALF – bronchoalveolar lavage fluid; BALT – bronchoalveolar lymphoid tissue; BrdU – 5-bromo-2-deoxyuridine; b.w. – body weight; CI, confidence interval; CC16 – Clara cell protein; ethylenediaminetetraacetic acid (EDTA; ELISA – enzyme-linked immunosorbent assay; ET-1 – endothelin-1; fCAP, fine concentrated ambient particulate matter;gsd – geometric standard deviation; GSH – reduced glutathione; GSSG – oxidized glutathione; HCT – haematocrit value; HDW – haemoglobin distribution width; HE – hematoxylin-eosin; HIA- Hendrik-Ido-Ambacht; LDH – lactate dehydrogenase; HGB – haemoglobin; HO-1, heme oxygenase; MDA, malondialdehyde; MIP-2 – macrophage inflammatory protein-2; MPC – mean platelet component; MPV – mean platelet volume; MCV – mean corpuscular volume; MCH – mean cell haemoglobin; MCHC – mean cell haemoglobin concentration; NAG – N-acetyl glucosaminidase; n.m., not measured; PM – particulate matter; PBS – phosphate buffered saline; PLT- platelet concentrations; PMN – polymorph nuclear neutrophil; PUF – polyurethane foam; RBC -red blood cell; RDW- red blood cell distribution width; SH – spontaneously hypertensive; TNF-α – tumour necrosis factor α; u+fCAP, ultrafine plus fine concentrated ambient particulate matter; UA – uric acid; vWF – von Willebrand factor; WBC- white blood cell.

## Competing interests

The author(s) declare that they have no competing interests.

## Authors' contributions

IMK has designed, coordinated and supervised the experimental work of this study, interpreted the results and drafted the manuscript. AJFB participated in the design and coordination of the study, carried out the in vivo experiments including sample handling, and participated in the statistical analysis. PHBF performed CAPs exposures and carried out the data analysis of the exposures. DLACL participated in the design, supported the in vivo experiments and collection of blood and tissue samples, carried out several BALF and blood analysis. JAMAD supported collection of lung tissue, performed histopathology and the statistical analysis for this part of the study. FRC is project leader, participated in conceiving the study, its design, interpretation of the results and is co-writer of the manuscript. All authors have read, reviewed, commented and approved the final manuscript.

**Figure 2:**
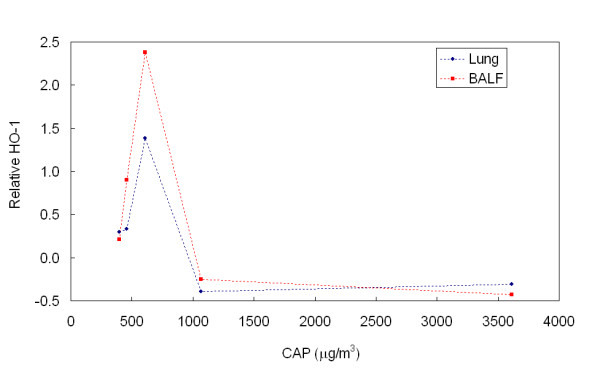
Relative heme oxygenase-1 present in bronchoalveolar lavage fluid () and in lung homogenate () versus the mass of fine, concentrated, ambient particulate matter. Relative heme oxygenase-1 is defined as ([HO-1]_CAP_- [HO-1]_Control_)/[HO-1]_Control_, where [HO-1] is the mean value of *n *= 8.

**Figure 3:**
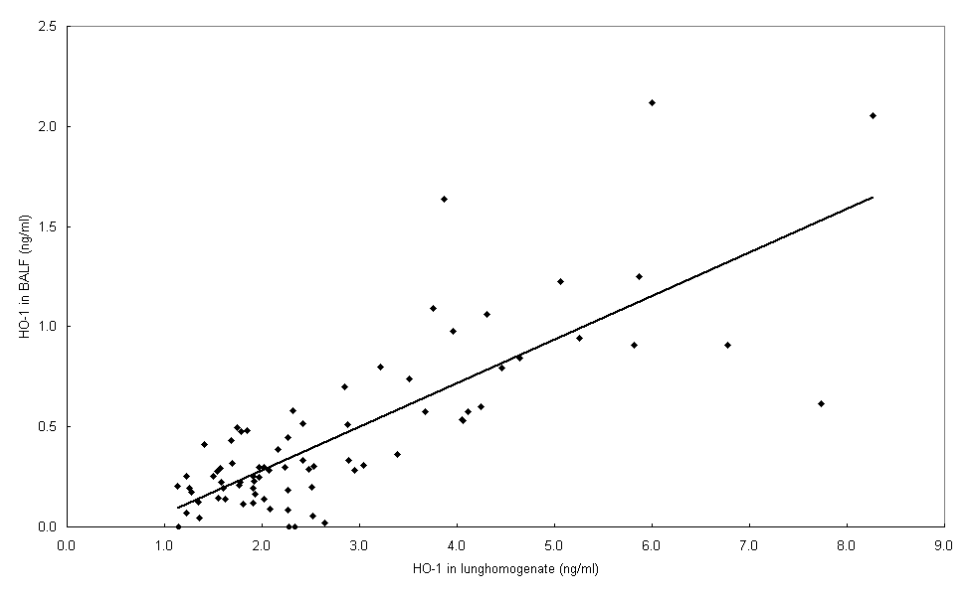
Correlation between heme oxygenase-1 present in bronchoalveolar lavage fluid versus heme oxygenase-1 present in lung homogenate. All animals were exposed to fine, concentrated, ambient particulate matter. Regression: Y = -0.15 + 0.217*X, correlation coefficient = 0.79.

**Figure 4:**
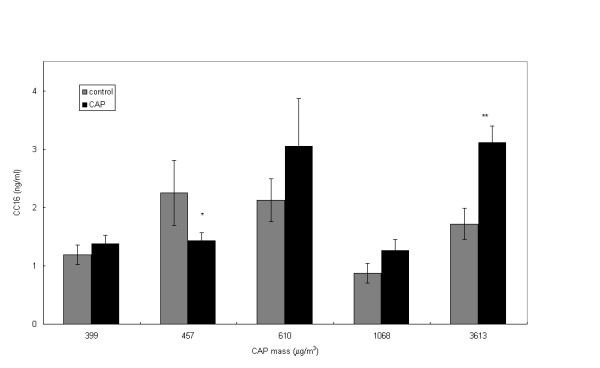
Clara cell secretory protein present in bronchoalveolar lavage fluid after rats were exposed to fine, concentrated, ambient particulate matter (fCAP) (site I)  control, ■ fCAP exposed.

**Figure 5:**
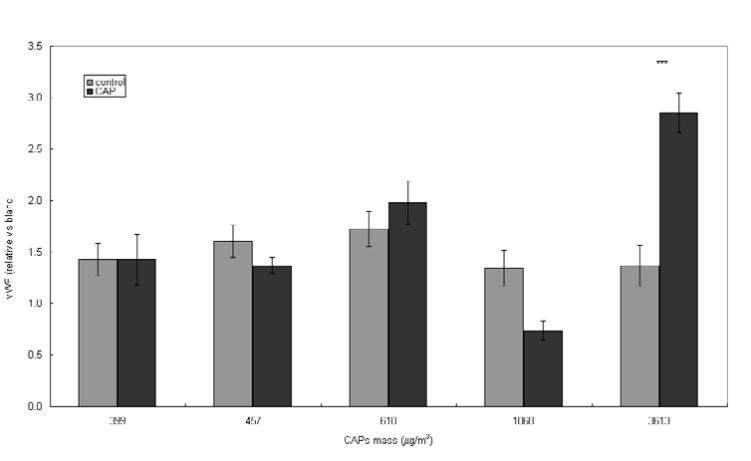
Von Willebrand factor present in citrate plasma after rats were exposed to fine, concentrated, ambient particulate matter (fCAP) (site I)  control, ■ fCAP exposed.

## References

[B1] Brunekreef B, Holgate ST (2002). Air pollution and health. Lancet.

[B2] Pope CAIII (2000). Epidemiology of fine particulate air pollution and human health: biologic mechanisms and who's at risk?. Environ Health Perspect.

[B3] Peters A, Dockery DW, Muller JE, Mittleman MA (2001). Increased particulate air pollution and the triggering of myocardial infarction. Circulation.

[B4] Samoli E, Analitis A, Touloumi G, Schwartz J, Anderson HR, Sunyer J, Bisanti L, Zmirou D, Vonk JM, Pekkanen J, Goodman P, Paldy A, Schindler C, Katsouyanni K (2005). Estimating the exposure-response relationships between particulate matter and mortality within the APHEA multicity project. Environ Health Perspect.

[B5] Katsouyanni K, Touloumi G, Samoli E, Gryparis A, Le Tertre A, Monopolis Y, Rossi G, Zmirou D, Ballester F, Boumghar A, Anderson HR, Wojtyniak B, Paldy A, Braunstein R, Pekkanen J, Schindler C, Schwartz J (2001). Confounding and effect modification in the short-term effects of ambient particles on total mortality: results from 29 European cities within the APHEA2 project. Epidemiology.

[B6] Dominici F, McDermott A, Zeger SL, Samet JM (2003). National maps of the effects of particulate matter on mortality: exploring geographical variation. Environ Health Perspect.

[B7] Dominici F, McDermott A, Daniels M, Zeger SL, Samet JM (2005). Revised analyses of the National Morbidity, Mortality, and Air Pollution Study: mortality among residents of 90 cities. J Toxicol Environ Health A.

[B8] Roberts S, Martin MA (2006). Applying a moving total mortality count to the cities in the NMMAPS database to estimate the mortality effects of particulate matter air pollution. Occup Environ Med.

[B9] Roemer WH, van Wijnen JH (2001). Daily mortality and air pollution along busy streets in Amsterdam, 1987-1998. Epidemiology.

[B10] Schlesinger RB, Cassee FR (2003). Atmospheric secondary inorganic particulate matter: the toxicological perspective as a basis for health effects risk assessment. Inhal Toxicol.

[B11] Sioutas C, Koutrakis P, Godleski J, Ferguson S, Kim C, Burton R (1997). Harvard/EPA ambient fine particle concentrators for human and animal exposures. J Aerosol Sci.

[B12] Kim S, Jaques P, Chang MC, Froines JR, Sioutas C (2001). A versatile aerosol concentrator for simultaneous in vivo and in vitro evaluation of toxic effects of  coarse, fine and ultrafine particiles: Part I: Laboratory evaluation. Jouran of Aerosol Science.

[B13] Kim S, Jaques P, Chang MC, Xiong C, Friedlander SK, Sioutas C (2001). A versatile aerosol concentrator for simultaneous in vivo and in vitro evaluation of toxic effects of  coarse, fine and ultrafine particiles: Part II: Field evaluation. Jouran of Aerosol Science.

[B14] Lippmann M, Gordon T, Chen LC (2005). Effects of subchronic exposures to concentrated ambient particles (CAPs) in mice. I. Introduction, objectives, and experimental plan. Inhal Toxicol.

[B15] Kodavanti UP, Schladweiler MC, Ledbetter AD, McGee JK, Walsh L, Gilmour PS, highfill JW, Davies D, Pinkerton KE, Richards JH, Crissman K, Andrews D, Costa DL (2005). Consistent pulmonary and systemic responses from inhalation of fine concentrated ambient particles: roles of rat strains used and physicochemical properties. Environ Health Perspect.

[B16] Harkema JR, Keeler G, Wagner J, Morishita M, Timm E, Hotchkiss J, Marsik F, Dvonch T, Kaminski N, Barr E (2004). Effects of concentrated ambient particles on normal and hypersecretory airways in rats. Res Rep Health Eff Inst.

[B17] Smith KR, Kim S, Recendez JJ, Teague SV, Menache MG, Grubbs DE, Sioutas C, Pinkerton KE (2003). Airborne particles of the california central valley alter the lungs of healthy adult rats. Environ Health Perspect.

[B18] Saldiva PH, Clarke RW, Coull BA, Stearns RC, Lawrence J, Murthy GG, Diaz E, Koutrakis P, Suh H, Tsuda A, Godleski JJ (2002). Lung inflammation induced by concentrated ambient air particles is related to particle composition
1. Am J Respir Crit Care Med.

[B19] Kodavanti UP, Schladweiler MC, Ledbetter AD, Watkinson WP, Campen MJ, Winsett DW, Richards JR, Crissman KM, Hatch GE, Costa DL (2000). The spontaneously hypertensive rat as a model of human cardiovascular disease: evidence of exacerbated cardiopulmonary injury and oxidative stress from inhaled emission particulate matter. Toxicol Appl Pharmacol.

[B20] Cassee FR, Boere AJ, Bos J, Fokkens PH, Dormans JA, van Loveren H (2002). Effects of diesel exhaust enriched concentrated PM2.5 in ozone preexposed or monocrotaline-treated rats. Inhal Toxicol.

[B21] Wellenius GA, Coull BA, Godleski JJ, Koutrakis P, Okabe K, Savage ST, Lawrence JE, Murthy GG, Verrier RL (2003). Inhalation of concentrated ambient air particles exacerbates myocardial ischemia in conscious dogs. Environ Health Perspect.

[B22] Devlin RB, Ghio AJ, Kehrl H, Sanders G, Cascio W (2003). Elderly humans exposed to concentrated air pollution particles have decreased heart rate variability. Eur Respir J Suppl.

[B23] Brook RD, Brook JR, Urch B, Vincent R, Rajagopalan S, Silverman F (2002). Inhalation of fine particulate air pollution and ozone causes acute arterial vasoconstriction in healthy adults. Circulation.

[B24] Ghio AJ, Kim C, Devlin RB (2000). Concentrated ambient air particles induce mild pulmonary inflammation in healthy human volunteers. Am J Respir Crit Care Med.

[B25] Ghio AJ, Hall A, Bassett MA, Cascio WE, Devlin RB (2003). Exposure to concentrated ambient air particles alters hematologic indices in humans. Inhal Toxicol.

[B26] Gong HJ, Linn WS, Sioutas C, Terrell SL, Clark KW, Anderson KR, Terrell LL (2003). Controlled exposures of healthy and asthmatic volunteers to concentrated ambient fine particles in los angeles. Inhal Toxicol.

[B27] Cassee FR, Boere AJ, Fokkens PH, Leseman DL, Sioutas C, Kooter IM, Dormans JA (2005). Inhalation of concentrated particulate matter produces pulmonary inflammation and systemic biological effects in compromised rats. J Toxicol Environ Health A.

[B28] Seagrave J, McDonald JD, Mauderly JL (2005). In vitro versus in vivo exposure to combustion emissions. Exp Toxicol Pathol.

[B29] Li N, Kim S, Wang M, Froines J, Sioutas C, Nel A (2002). Use of a stratified oxidative stress model to study the biological effects of ambient concentrated and diesel exhaust particulate matter. Inhal Toxicol.

[B30] Oberdorster G, Ferin J, Gelein R, Soderholm SC, Finkelstein J (1992). Role of the alveolar macrophage in lung injury: studies with ultrafine particles. Environ Health Perspect.

[B31] Hetland RB, Cassee FR, Refsnes M, Schwarze PE, Lag M, Boere AJ, Dybing E (2004). Release of inflammatory cytokines, cell toxicity and apoptosis in epithelial lung cells after exposure to ambient air particles of different size fractions. Toxicol In Vitro.

[B32] Oberdorster G (1995). Lung particle overload: implications for occupational exposures to particles. Regul Toxicol Pharmacol.

[B33] Peters A, Doring A, Wichmann HE, Koenig W (1997). Increased plasma viscosity during an air pollution episode: a link to mortality?. Lancet.

[B34] Ibald-Mulli A, Wichmann HE, Kreyling W, Peters A (2002). Epidemiological evidence on health effects of ultrafine particles. J Aerosol Med.

[B35] Timonen KL, Hoek G, Heinrich J, Bernard A, Brunekreef B, de Hartog J, Hameri K, Ibald-Mulli A, Mirme A, Peters A, Tiittanen P, Kreyling WG, Pekkanen J (2004). Daily variation in fine and ultrafine particulate air pollution and urinary concentrations of lung Clara cell protein CC16. Occup Environ Med.

[B36] de Hartog JJ, Hoek G, Peters A, Timonen KL, Ibald-Mulli A, Brunekreef B, Heinrich J, Tiittanen P, van Wijnen JH, Kreyling W, Kulmala M, Pekkanen J (2003). Effects of fine and ultrafine particles on cardiorespiratory symptoms in elderly subjects with coronary heart disease: the ULTRA study. Am J Epidemiol.

[B37] Penttinen P, Timonen KL, Tiittanen P, Mirme A, Ruuskanen J, Pekkanen J (2001). Number concentration and size of particles in urban air: effects on spirometric lung function in adult asthmatic subjects. Environ Health Perspect.

[B38] Seaton A, MacNee W, Donaldson K, Godden D (1995). Particulate air pollution and acute health effects. Lancet.

[B39] Hahn FF, Barr EB, Menache MG, Seagrave J (2005). Particle size and composition related to adverse health effects in aged, sensitive rats. Res Rep Health Eff Inst.

[B40] Sorensen M, Daneshvar B, Hansen M, Dragsted LO, Hertel O, Knudsen L, Loft S (2003). Personal PM2.5 exposure and markers of oxidative stress in blood. Environ Health Perspect.

[B41] Rhoden CR, Lawrence J, Godleski JJ, Gonzalez-Flecha B (2004). N-acetylcysteine prevents lung inflammation after short-term inhalation exposure to concentrated ambient particles. Toxicol Sci.

[B42] Medina-Navarro R, Lifshitz A, Wacher N, Hicks JJ (1997). Changes in human serum antioxidant capacity and peroxidation after four months of exposure to air pollutants. Arch Med Res.

[B43] Otterbein LE, Bach FH, Alam J, Soares M, Tao LH, Wysk M, Davis RJ, Flavell RA, Choi AM (2000). Carbon monoxide has anti-inflammatory effects involving the mitogen-activated protein kinase pathway. Nat Med.

[B44] Chae HJ, Chin HY, Lee GY, Park HR, Yang SK, Chung HT, Pae HO, Kim HM, Chae SW, Kim HR (2005). Carbon monoxide and nitric oxide protect against tumor necrosis factor-alpha-induced apoptosis in osteoblasts: HO-1 is necessary to mediate the protection. Clin Chim Acta.

[B45] Keyse SM, Tyrrell RM (1989). Heme oxygenase is the major 32-kDa stress protein induced in human skin fibroblasts by UVA radiation, hydrogen peroxide, and sodium arsenite. Proc Natl Acad Sci U S A.

[B46] Li N, Venkatesan MI, Miguel A, Kaplan R, Gujuluva C, Alam J, Nel A (2000). Induction of heme oxygenase-1 expression in macrophages by diesel exhaust particle chemicals and quinones via the antioxidant-responsive element.. J-Immunol.

[B47] Li N, Sioutas C, Cho A, Schmitz D, Misra C, Sempf J, Wang M, Oberley T, Froines J, Nel A (2003). Ultrafine particulate pollutants induce oxidative stress and mitochondrial damage. Environ Health Perspect.

[B48] Gordon T, Nadziejko C, Schlesinger R, Chen LC (1998). Pulmonary and cardiovascular effects of acute exposure to concentrated ambient particulate matter in rats. Toxicol Lett.

[B49] Nadziejko C, Fang K, Chen LC, Cohen B, Karpatkin M, Nadas A (2002). Effect of concentrated ambient particulate matter on blood coagulation parameters in rats. Res Rep Health Eff Inst.

[B50] Ulrich MM, Alink GM, Kumarathasan P, Vincent R, Boere AJ, Cassee FR (2002). Health effects and time course of particulate matter on the cardiopulmonary system in rats with lung inflammation. J Toxicol Environ Health A.

[B51] Gilmour PS, Ziesenis A, Morrison ER, Vickers MA, Drost EM, Ford I, Karg E, Mossa C, Schroeppel A, Ferron GA, Heyder J, Greaves M, MacNee W, Donaldson K (2004). Pulmonary and systemic effects of short-term inhalation exposure to ultrafine carbon black particles. Toxicol Appl Pharmacol.

[B52] Huang YC, Ghio AJ, Stonehuerner J, McGee J, Carter JD, Grambow SC, Devlin RB (2003). The role of soluble components in ambient fine particles-induced changes in human lungs and blood. Inhal Toxicol.

[B53] Kodavanti UP, Schladweiler MC, Ledbetter AD, Hauser R, Christiani DC, McGee J, Richards JR, Costa DL (2002). Temporal association between pulmonary and systemic effects of particulate matter in healthy and cardiovascular compromised rats. J Toxicol Environ Health A.

[B54] Sela S, Mazor R, Amsalam M, Yagil C, Yagil Y, Kristal B (2004). Primed polymorphonuclear leukocytes, oxidative stress, and inflammation antecede hypertension in the Sabra rat. Hypertension.

[B55] Seaton A, Soutar A, Crawford V, Elton R, McNerlan S, Cherrie J, Watt M, Agius R, Stout R (1999). Particulate air pollution and the blood. Thorax.

[B56] Macey MG, Carty E, Webb L, Chapman ES, Zelmanovic D, Okrongly D, Rampton DS, Newland AC (1999). Use of mean platelet component to measure platelet activation on the ADVIA 120 haematology system. Cytometry.

